# A matter of time: improvement of visual temporal processing during training-induced restoration of light detection performance

**DOI:** 10.3389/fpsyg.2015.00022

**Published:** 2015-02-11

**Authors:** Dorothe A. Poggel, Bernhard Treutwein, Bernhard A. Sabel, Hans Strasburger

**Affiliations:** ^1^Generation Research Program, Human Science Center, Ludwig-Maximilian University, Bad Tölz, Germany; ^2^Hanse-Wissenschaftskolleg Institute for Advanced Study, Neurosciences and Cognitive Sciences, Delmenhorst, Germany; ^3^IuK, Ludwig-Maximilian University, Munich, Germany; ^4^Institute of Medical Psychology, Medical Faculty, Otto-von-Guericke University, Magdeburg, Germany; ^5^Department of Medical Psychology and Medical Sociology, Georg-August University, Göttingen, Germany; ^6^Institute of Medical Psychology, Ludwig-Maximilian University, Munich, Germany

**Keywords:** blindness, temporal resolution, reaction time, visual restoration, training, plasticity, visual field, topography

## Abstract

The issue of how basic sensory and temporal processing are related is still unresolved. We studied temporal processing, as assessed by simple visual reaction times (RT) and double-pulse resolution (DPR), in patients with partial vision loss after visual pathway lesions and investigated whether vision restoration training (VRT), a training program designed to improve light detection performance, would also affect temporal processing. Perimetric and campimetric visual field tests as well as maps of DPR thresholds and RT were acquired before and after a 3 months training period with VRT. Patient performance was compared to that of age-matched healthy subjects. Intact visual field size increased during training. Averaged across the entire visual field, DPR remained constant while RT improved slightly. However, in transition zones between the blind and intact areas (areas of residual vision) where patients had shown between 20 and 80% of stimulus detection probability in pre-training visual field tests, both DPR and RT improved markedly. The magnitude of improvement depended on the defect depth (or degree of intactness) of the respective region at baseline. Inter-individual training outcome variability was very high, with some patients showing little change and others showing performance approaching that of healthy controls. Training-induced improvement of light detection in patients with visual field loss thus generalized to dynamic visual functions. The findings suggest that similar neural mechanisms may underlie the impairment and subsequent training-induced functional recovery of both light detection and temporal processing.

## INTRODUCTION

Visual signals contain information on many different aspects of our environment. Most prominently, intensity (or contrast), spatial configuration, and temporal aspects are important dimensions of visual perception. Traditionally, research on temporal aspects of perception was either concerned with higher cognitive mechanisms, for instance the estimation of interval duration—which we term “time perception”—or it examined basic psychophysical aspects of temporal parameters and their connection with basic perceptual functions (Wittmann, 1999, 2009)—which we term “temporal processing.” In the study presented here we are exclusively concerned with the latter.

The two major aspects of temporal processing are the speed of visual perception as such—which can be measured, for instance, by simple visual reaction times (RTs; as explained in the methods section)—and the temporal resolution of visual perception—which can be measured for example by flicker resolution tasks. These two aspects in the temporal domain (“*when* do I perceive?” and “*how fine-grain* is the perception?”) correspond basically to analogous concepts in the spatial domain (“where do I perceive?”—i.e., localization tasks—and “how fine-grain is the spatial resolution?”—i.e., can the perceiver discriminate between location A and B and what is the minimal distance between A and B that still allows that discrimination).

So far, there is no agreed-upon theoretical framework that may explain how different dimensions of visual processing (intensity, space, time) are related on the neural level: for example, it is not fully understood how they are integrated into a coherent percept, though neural synchronization seems to be involved (e.g., Singer and Gray, 1995). Particularly, the mechanisms underlying processing of time-related information in the brain and their interactions with early sensory processes are still poorly understood (Ivry and Spencer, 2004; Mauk and Buonomano, 2004; Poggel and Strasburger, 2004; Poggel et al., 2012a,b; see Wittmann, 1999, 2009 for a review).

Numerous studies have provided evidence for close connections between visual stimulus intensity and temporal visual functions, e.g., using RTs or flicker detection (Kelly, 1972; Ulrich et al., 1998). However, most of these studies suffer from methodological shortcomings: in many cases, measurements were limited to the fovea and thus neglected the spatial dimension of vision and characteristics of the peripheral visual field (Poggel and Strasburger, 2004; Strasburger et al., 2011; Poggel et al., 2012a,b). Moreover, the employed flicker detection tasks were dependent on adaptation and modulation depth (Tyler, 1985, 1987; Treutwein, 1989; Tyler and Hamer, 1990; Treutwein and Rentschler, 1992).

In a normative study with a large sample of healthy subjects (Poggel et al., 2012a,b), we therefore took all three dimensions into consideration: stimulus intensity (by measuring perimetric luminance thresholds), spatial aspects (by performing measurements across the visual field), and temporal aspects (by measuring RTs and temporal resolution independently of the modulation depth). Interestingly, there was a clear dissociation between perimetric thresholds, RTs, and temporal resolution thresholds: not only did the maps of these three variables show different topographies, but there was also dissociation across the life span, i.e., the three variables showed different topographical patterns of aging. It thus seems that, as explained above, RTs and temporal resolution are based on different neural mechanisms: while (simple) RTs mainly depend on the speed of neural transmission (through the visual system and subsequently the motor system), temporal resolution can be assumed to depend on a read-out mechanism for separating two bursts of action potentials (corresponding to the two light pulses), the success of which depends on the degree of overlap between the first and second burst and thus on the signal-to-noise ratio rather than on the speed of transmitting the activation along the visual pathway (see Figure 6 in Poggel et al., 2006). Furthermore, the relationship between intensity measures (like light detection thresholds and contrast thresholds), and RTs or temporal resolution in the periphery of the visual field is not predicted by their relationship when measured solely in the fovea (as is done in most studies in the literature).

To further investigate potential connections or dissociations between visual and temporal functions, we looked at patients with vision loss resulting from lesions of the visual pathway. Experimental evidence (Strasburger and Rentschler, 1996; Gothe et al., 2000; Bola et al., 2014) as well as subjective complaints of patients (Poggel, 2002) had pointed earlier to a topographic mismatch between perimetric thresholds (the gold standard in clinical testing) and other visual and temporal functions, e.g., RTs, that do not play a role in standard clinical testing. When we investigated a patient sample (Poggel et al., 2011) with the same methods as in the normative study mentioned above (Poggel et al., 2012a,b), we found deficits of temporal processing (RTs and temporal resolution thresholds) across the entire visual field, i.e., even in areas that were perimetrically intact. Furthermore, performance of temporal processing within the defective visual field depended on the degree of intactness (or defect depth) of the respective visual field location. Thus, damage to the visual pathway also affects temporal processing of visual stimuli, and to a certain extent those deficits do not correspond with maps of perimetric light detection performance.

The overlap or dissociation of visual function maps is not only of interest for elucidating basic mechanisms of visual integration or for the planning of diagnostic procedures, but it is also clinically relevant with respect to processes of visual brain plasticity and treatment of vision loss. Studies on perceptual learning in healthy populations (Fine and Jacobs, 2002; Seitz and Watanabe, 2005; Jüttner and Rentschler, 2008; Gilbert et al., 2009; Fahle, 2009) and also clinical studies with visually impaired patients (van der Wildt and Bergsma, 1997; Kasten et al., 1998; Kerkhoff, 1999; Sabel, 1999, 2008; Poggel, 2002; Julkunen et al., 2003; Poggel et al., 2004; Sahraie, 2007; Huxlin, 2008; Bergsma and van der Wildt, 2010; Sabel and Gudlin, 2014) have demonstrated training-induced improvement of function, particularly of light detection performance (see Sabel et al., 2011, for a review). Perceptual learning experiments in healthy subjects have shown that the observed improvements are often specific to a visual function or to the visual field region targeted by the training (Fine and Jacobs, 2002; Fahle, 2009; Strasburger et al., 2011). Similarly, although previous light-detection training studies with patients showed some generalization to other functions like color and form discrimination (Kasten and Sabel, 1995; Kasten et al., 2000), a specific training of that particular function had a much more pronounced effect (Poggel, 2002).

The findings of an overlap as well as dissociations between light detection and temporal processing functions in healthy populations and in patients—but also previous evidence for at least some generalization in perceptual learning and training-induced recovery of visual function—led us to ask whether and to what extent vision restoration training (VRT) targeted at recovery of light detection would also have beneficial effects on temporal processing in patients with visual field loss. The potential benefits of this study would be twofold: on the one hand, finding “positive side effects” of light-detection training on dynamic visual functions would be of direct use to patients complaining about difficulties with dynamic vision; on the other hand, from a basic science perspective, the findings would provide a basis for investigating whether or not the intensity and the temporal aspect of vision may have a common neural basis.

## MATERIALS AND METHODS

### PATIENT SAMPLE AND HEALTHY CONTROL GROUP

Nine patients with visual field loss participated in the study (three female; mean age 42 years ± 4.5 years, range 22–62 years; Table 1).

**Table 1:** **Patient characteristics**.

Patient number	Age (years)	Gender (female/male)	Lesion age (months)	Hemisphere (left/right)	Location of lesion	Cause of lesion	Vision loss
2	35	Female	60	Right	Posterior artery	Aneurysm clipping	Hom. hemianopia left
3	62	Female	27	Right	Medial artery (?)	Infarction	Hom. hemianopia left
4	49	Male	36	Optic nerve	Optic nerve	Tumor surgery	Bilateral, heteronymous
5	43	Male	11	Right	Optic radiation	Infarction	Hom. quadrantanopia
6	22	Female	8	Right	Posterior artery	Infarction	Hom. quadrantanopia
7	44	Male	87	Right	Posterior artery	Infarction	Hom. hemianopia
8	23	Male	42	Left	Medial artery	Trauma	Hom. hemianopia
9	51	Male	10	Left	Posterior artery (?)	Bleeding	Hom. hemianopia
12	44	Male	27	Right	Posterior artery (?)	Infarction	Hom. hemianopia

Exclusion criteria for the study were dementia, hemispatial neglect, severe attentional deficits (especially reduced vigilance), depression and other psychiatric disorders, as well as visual impairment resulting from ophthalmic diseases. All subjects gave their informed consent for participation in the study. The experimental design had been approved by the local ethics committee and was in accordance with the guidelines of the Declaration of Helsinki.

Patients’ performance was compared to normative data from 95 healthy participants who had been tested with the same set of methods in the Tölz Temporal Topography Study (Poggel et al., 2012a,b).

Patients served as their own control group: only patients with chronic, stable vision loss were included in this study. Stability of visual field size was ascertained by repeated visual field testing over a period of several weeks or months before and after training. Since the effectiveness of the training program had been shown earlier in two randomized, placebo-controlled trials (Kasten et al., 1998; Sabel and Gudlin, 2014), we did not include a placebo control group here.

### DOUBLE-PULSE RESOLUTION

For assessing temporal resolution in the visual field, we measured double-pulse resolution thresholds (DPR; Treutwein, 1989, 1995, 1997; Treutwein and Rentschler, 1992). Participants were sitting in a darkened room (illuminance 1.5 lx), their head positioned on a chin rest at 30 cm viewing distance in front of a test screen. Stimuli were presented with microsecond accuracy on a 17-inch *x*-*y*-*z* monitor (HP 1310) that was controlled by D/A converters (“point plot buffer”; G. Finlay, Edmonton, Canada) connected to a PC.

A cross-hair was displayed before each trial. During a trial, nine rectangular white light stimuli (luminance: 215 cd/m^2^, size: 1.15°) were presented simultaneously on the screen, one in the center, and the others on a circle around it at the intersections with the main horizontal, vertical, and 45° meridians. Eight of the nine stimuli within a trial served as distracters and were presented continuously, while the target was interrupted by a temporal gap which resulted in the perception of a short flicker of that stimulus for gap durations above threshold. For each trial, the participant verbally indicated the target position, and the experimenter entered the response using the computer keyboard so that the participant could keep their eyes fixated at the center of the screen. Fixation was controlled with an eye tracking device (IViewX, Sensomotoric Instruments, Teltow, Germany) and by the experimenter observing the subject’s eye position via a mirror. The new trial was started when the subject was ready with stable fixation at the center of the screen.

The YAAP maximum-likelihood algorithm (Treutwein, 1995, 1997) controlled the gap duration between the two light pulses of the target stimulus. The starting point was set to 80 ms which was well above threshold for intact positions in the visual field. DPR thresholds were determined independently of each other in an interleaved fashion; target positions varied randomly from trial to trial. For stabilizing the adaptive procedure, the first 10 trials were non-adaptively presented according to the method of constant stimuli and an *a priori* distribution was created by calculating the likelihoods for these responses. These responses were included in the final estimates. Guessing resulted in an *a priori* ceiling value of >100 ms at the blind locations in the visual field. The first light pulse of the target stimulus had 80 ms duration, the second (after the gap) 280 ms (see Treutwein, 1989; Treutwein and Rentschler, 1992, for details on stimulus parameters). The distracters were presented simultaneously with the target so that their duration matched that of the complete target stimulus including the gap. Note that targets and non-targets appeared equal in brightness since they were well above the summing duration in Bloch’s law (Treutwein, 1989; Treutwein and Rentschler, 1992).

A test block was ended when all nine thresholds were determined to a pre-specified confidence interval containing the threshold at 85% probability which took approximately 140–280 trials (between 10 and 20 min test duration). Eight blocks of trials were performed per subject. Within a block, the eccentricity of the peripheral stimuli, i.e., the ring radius, was constant. Four blocks were carried out with ascending ring radius of 2.5°, 5°, 10°, and 20°, respectively, followed by another four blocks in reverse order of eccentricities to balance series effects. Each eccentricity block thus occurred twice. DPR threshold maps were created by combining the results from test blocks of four eccentricities into an interpolated map (see below).

### LIGHT DETECTION AND REACTION TIME MAPS

Visual field maps were acquired for each eye separately using conventional static perimetry (Octopus 101 Perimeter, Interzeag/Haag Streit, Koeniz-Berne, Switzerland). Subsequently, a high-resolution computer-based campimetric test (HRP, Nova Vision GmbH, Magdeburg; see Kasten et al., 1997) was used for the acquisition of detailed light detection maps and RT maps under the same standardized conditions described above for DPR testing. A PC with a 17′ screen (horizontal size: ±29°, vertical size: ±23°, background luminance: 26 cd/m^2^) was used for presentation of the stimuli (circular white, luminance: 96 cd/m^2^, size: 0.76° visual angle, duration: 150 ms). Viewing was binocular in all patients except in the subject with optic nerve lesion who was tested on his left eye only. Stimuli were presented in random sequence at 474 positions on the screen. The fixation mark was positioned on the screen such that about half of the stimuli were situated in the blind field. The subject pressed the space bar on the computer keyboard whenever a stimulus was detected. Feedback of correct responses and false alarms, respectively, was provided by a high vs. low tone following the response. Stable fixation was ascertained by requiring the subject to detect a change of the fixation point’s color from equiluminant green to yellow (Kasten et al., 1997). Additionally, the eye position was recorded with an eye-tracker (see above), and it was monitored by the experimenter via a mirror.

Detected and missed stimuli were both registered by the test and mapped by the software. For detected stimuli, the RT was recorded. Results from five high-resolution campimetric tests were superimposed. This allowed computing detection probabilities at each location so that areas of residual vision near the border of the blind area with a stimulus detection rate between 20 and 80% could be mapped (see Poggel, 2002; Poggel et al., 2004). Subregions of areas of residual vision with 20, 40, 60, and 80% detection rate, respectively, were further outlined to reflect the defect depth or degree of impairment. RTs were averaged separately for each subregion. The same categorization was also used for comparison of DPR thresholds between regions with varying degree of lesion.

### TRAINING PROCEDURE

Based on the size and location of the areas of residual vision, each patient received an individualized training program (VRT, Nova Vision, Magdeburg, Germany) that provided stimulation focused on the border of the defect, i.e., on the areas having the largest probability of training-induced improvement (Kasten et al., 1998; Poggel et al., 2004, 2008). Stimulus size, fixation control, and response procedures were identical to those of the HRP visual field test described above. Training stimuli appeared on the computer screen, increasing in brightness over a period of 2000 ms. Each training session lasted approximately 15–20 min and comprised 250 training stimuli. The patient performed three training units of 56 sessions each, so that one training unit was completed in about one calendar month if the patient complied with the recommended two sessions per day. The training software provided feedback on the number of stimuli detected after each session. After each training unit, the patient returned to the laboratory for a control examination consisting of a short interview, a visual field test, and the analysis of the training data, followed by an adjustment of the training area to accommodate any progress the patient had made. After the third training unit, post-training measurements were performed which were essentially identical to the pre-training baseline examinations described above.

### DATA ANALYSIS

Each DPR test block with a specific eccentricity of the peripheral test location was presented twice: once in a sequence of ascending eccentricities and the second time in a sequence of descending eccentricities over test blocks. There was no significant difference between the DPR threshold values from the first and second test at the corresponding eccentricities. Therefore, the respective test results were averaged to increase reliability.

Raw data from DPR, campimetric, and perimetric tests, respectively, were entered into statistical software for data analysis (Microsoft Excel and SPSS Version 15, Chicago, IL, USA) and subsequently plotted with a Matlab script (see Gothe et al., 2000), with linear interpolation between average values at all target positions (Matlab Version 5.3, The MathWorks, Natick, MA, USA).

To determine the influence of eccentricity on performance, we calculated the averages over all test positions for a specific ring (i.e., test eccentricity). For a global comparison between subjects, the overall average over all visual field positions was determined per subject, as well as individual performance in the defective and intact hemifield (note that there were some intact or partially intact positions remaining in the defective hemifield so that these values could be calculated). For the topographical comparison of DPR and RTs, we matched the less densely sampled DPR positions to those in campimetric tests, and selected for analysis only the RT values at corresponding positions. These values were averaged and imported into Matlab for plotting. For a topographical comparison between DPR and RTs within subjects, we calculated, for each patient, the correlations between the two variables at corresponding visual field locations, and these topographical correlations were then averaged across subjects.

For each of the subregions of areas of residual vision (20–80% detection rate in five campimetric tests), we next calculated average DPR thresholds and average RTs. RT values of all five campimetric tests were averaged. Note that any variation of RTs across the visual field reflects the sensory component only (including decisions on sensory data), since motor requirements are invariant, i.e., contribute only to the absolute level of RTs (Teichner and Krebs, 1972; Schiefer et al., 2001). Patient DPR and RTs were further compared to normative data of the respective age group of each patient.

Non-parametric tests were used to compare average values (Kruskal–Wallis test, Mann–Whitney *U*-test, Wilcoxon test) and to test for the significance of correlations (Spearman’s Rho). With the small sample size of our patient group and for the comparisons between healthy participants and patients with differences in sample size, we preferred non-parametric statistics as the more appropriate way of testing in these cases. For the within-subjects comparisons between different eccentricities and between areas with different defect depth (i.e., detection probability at baseline), we used parametric testing with caution to be able to compare the averages, e.g., in the *post hoc* comparisons. RT data were analyzed with parametric methods (*t*-test for comparison of averages and Pearson’s coefficient (*r*) for correlations). For multiple comparisons between or within subjects, ANOVAs were employed. All statistical testing was done with SPSS (Version 15.0, Chicago, IL, USA). The alpha-level was set to 0.05, two-tailed.

## RESULTS

### IMPROVEMENT OF LIGHT DETECTION PERFORMANCE

**FIGURE 1:**
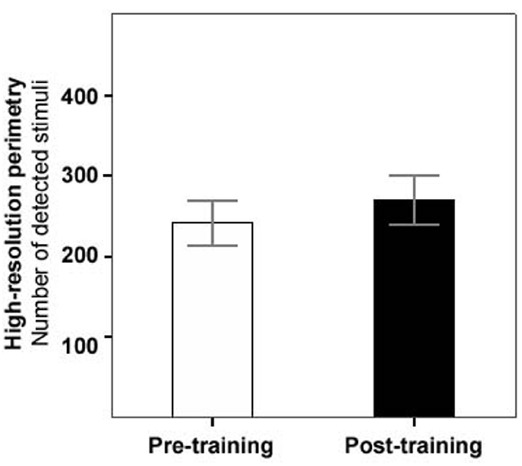
**Increase of light detection performance before vs. after training.** Mean number of detected stimuli (out of 474) in computer-based high-resolution perimetry before (white bar) and after training (black bar), across the patient group (error bars represent SEM).

During the 3-month training period, the patient group improved slightly but significantly in their average light detection performance. The average number of detected light stimuli in the computer-based campimetric visual field test (HRP) increased from 247.5 (±25.8 SEM) to 272.9 (±26.5) stimuli (Wilcoxon test: *Z* = 1.96, *p* = 0.05; *t*-test: *t* = 2.49, *p* = 0.01; Figure 1). In the conventional perimetric test (Oculus), the overall number of absolute defects (no detection) and relative defects (detection with increased threshold) in the visual field decreased over treatment, which was significant for the average number of absolute defects on the right eye only, however (before training: 44.8 ± 6.2, after training: 34.3 ± 7.1, Wilcoxon test: *Z* = 2.52, *p* = 0.01).

**FIGURE 2:**
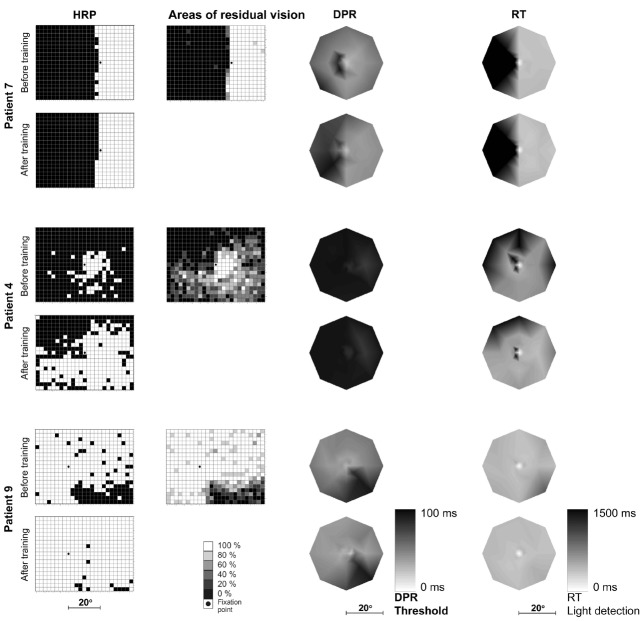
**Topography of light detection, DPR, and RTs before and after training.** Typical examples of three patients with different magnitudes of change: no improvement (patient 7; first two rows), intermediate success (patient 4; middle two rows), and strong recovery (patient 9; bottom two rows) over 3 months of vision restoration training. For each patient, light detection performance in high-resolution perimetry (HRP; leftmost column) is shown before and after training. Black: blind field, white: intact field. The second column shows areas of residual vision (or transition zones) before training: shades of gray represent the probability of stimulus detection at each location. Double-pulse resolution (DPR) thresholds (third column) are plotted for the inner 20° radius of the visual field, before and after training (lighter areas represent better temporal resolution, i.e., lower thresholds). RTs in response to simple light stimuli in HRP before and after training (right column) are shown for the same visual field positions as for DPR measurements (lighter areas represent faster responses). Note that DPR and RT plots are shown in central fixation perspective while the visual field (detection) maps in the first two columns show the fixation position on the screen as presented in the original test.

As expected from earlier studies, the variation of improvement between patients was large: several patients showed no improvement at all whereas others showed a strong treatment effect and a marked increase of intact areas. Patient 7 with a complete hemianopia and almost no areas of residual vision, for example, showed an unchanged visual field border before vs. after training. Patient 4 showed an intermediate (but statistically significant) success of visual field increase. Patient 9 with an incomplete quadrantanopia and large areas of residual vision had an almost intact visual field after training with respect to light detection (Figure 2).

### IMPROVEMENT OF TEMPORAL RESOLUTION (DPR THRESHOLDS)

**FIGURE 3:**
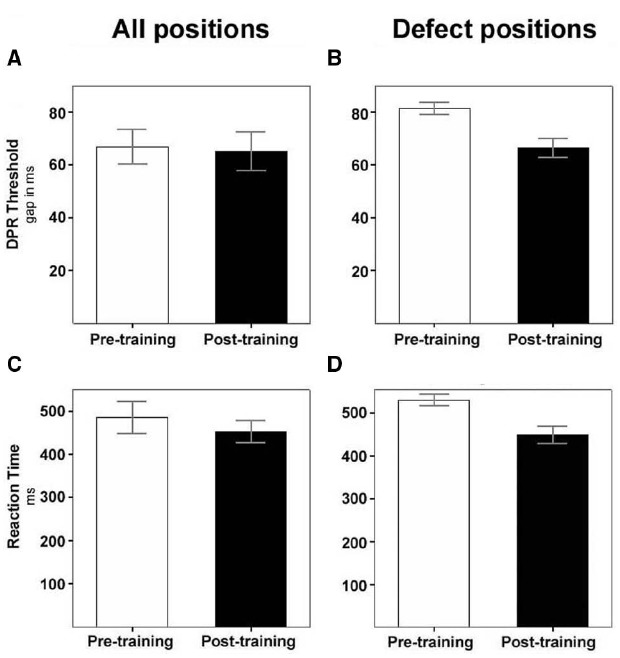
**Decrease of DPR thresholds and of RTs over training.** White bars, before training; black bars, after training. **(A)** Mean DPR thresholds (±SEM) of the total patient sample for all visual field positions, including intact areas; **(B)** mean DPR thresholds (±SEM) of the total patient sample for positions in the defective field only. **(C)** Mean RTs (±SEM) of the total patient sample for all visual field positions, including intact areas; (**D)** mean RTs (±SEM) of the total patient sample for positions in the defective field only.

The group-mean DPR threshold over the entire visual field showed high variance and did not significantly change over the training period (DPR pre-training: 66.8 ± 6.6 ms, DPR post-training: 65.3 ± 7.4 ms; Wilcoxon test: *Z* = 0.84, *p* = 0.40; Figure 3A). However, when DPR thresholds in just the defective parts of the visual field (the hemifield or quadrant(s) containing the blind area) were compared, we found highly significant improvements (pre-training: 81.4 ms ± 2.4, post-training: 66.5 ms ± 3.7, Wilcoxon *Z* = 2.64, *p* = 0.008; Figure 3B).

**FIGURE 4:**
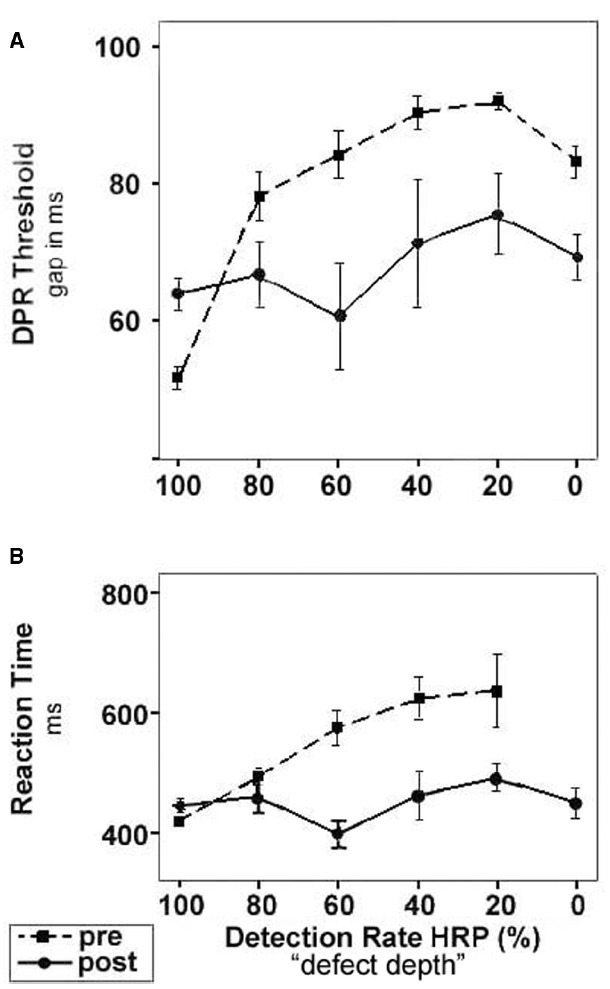
**DPR threshold and RT improvement depends on defect depth of visual field region.** Dashed lines with square symbols: before training; solid lines with circle symbols, after training. Categorization of visual field regions was based on pre-training baseline measurements: areas with 100% detection rate were considered intact; areas of 0% detection probability were considered blind. Regions of intermediate detection performance of 20–80% were defined as areas of residual vision. **(A) **DPR thresholds before and after training plotted as mean (±SEM) over visual field regions with different defect depth. The most intense improvement of DPR thresholds was found in areas of residual vision. **(B)** RTs before and after training plotted as mean (±SEM) over visual field regions with different defect depth. The largest reduction of RTs was observed in areas of residual vision. Note that RT cannot be determined in blind areas. After training, RTs could be measured in areas which had been blind at baseline and which had partially recovered.

The improvement of DPR thresholds did not depend on eccentricity (MANOVA: df = 4, *F* = 0.32; *p* = 0.86) but was instead influenced by the *degree of intactness* (or defect depth) of the respective position stimulated during treatment (MANOVA: df = 5, *F* = 14.80; *p* < 0.001). Particularly partially lesioned visual field areas (i.e., with pre-training detection rates between 20 and 80%)—which were at the same time the regions with the most prominent increase of light detection performance—showed the most pronounced reduction of DPR thresholds (Figure 4A).

Again, the variation of training effects between patients was considerable. Interestingly, the effects on temporal resolution and their topography were related to those of light detection, i.e., patients who improved in light detection typically also showed a decrease of DPR thresholds, and the improvements took place in roughly the same visual field locations (Figure 2). Conversely, patient 7 who showed no change of light detection performance (see Figure 2) also did not improve with respect to DPR thresholds (DPR pre: 46.9 ± 2.4 ms, DPR post: 49.9 ± 2.5 ms; Wilcoxon test: *Z* = 0.95, *p* = 0.34). Accordingly, there was no change in his DPR performance map as a result of training. Compared to healthy subjects of his age group he had normal DPR thresholds before and after training in his intact area. In contrast, patient 4 had markedly elevated DPR thresholds compared to his healthy age-matched control group, both before and after training. He improved only slightly (but not significantly) with respect to temporal resolution over the training period (DPR pre 92.1 ± 0.8 ms, DPR post: 90.9 ± 0.9 ms; Wilcoxon test: *Z* = 1.40, *p* = 0.16), i.e., there was a considerable dissociation of light detection and DPR threshold maps after training. Patient 9, who showed a strong improvement of light detection in the lower right quadrant (Figure 2) also improved significantly with respect to DPR thresholds (DPR pre: 48.2 ± 2.2., DPR post: 44.7 ± 2.1; Wilcoxon test: *Z* = 2.20, *p* = 0.03). DPR thresholds for this patient reached a normal level after training, both in the intact and in the previously defective visual field.

Before training, mean DPR thresholds (i.e., averaged across all visual field positions) were significantly higher for patients than for a sample of healthy subjects of all age groups (DPR-pre patients: 62.2 ± 1.7 ms, DPR healthy: 50.4 ± 0.9 ms, Mann–Whitney test: *Z* = 9.53, *p* < 0.001). Compared to the normally sighted controls, particularly, DPR thresholds were elevated in the patients’ defective region of the visual field, but several patients also had increased thresholds even in perimetrically intact areas (see Poggel et al., 2011). After training, the difference of DPR thresholds between the patients and the healthy controls was significantly reduced. However, even after treatment, patients’ DPR thresholds were still elevated compared to the healthy sample, although to a lesser extent (DPR-post patients: 61.7 ± 1.8 ms; Mann–Whitney test: *Z* = 7.89, *p* < 0.001). Again, the individual response varied: while several patients did not reach normal levels of temporal resolution even after training, other patients were within the range of their age-matched healthy controls even before training.

### IMPROVEMENT OF SIMPLE VISUAL REACTION TIMES

RTs to simple light stimuli presented in the high-resolution campimetric test decreased by ∼30 ms on average in the patient group over the period of training, although the difference missed significance due to the high variance between patients (RT pre-training ± SEM: 484.8 ± 37.6 ms, RT-post: 452.4 ± 26.5 ms; Wilcoxon test: *Z* = 1.68, *p* = 0.093; Figure 3C). Again, the improvement of RTs was much more pronounced (82 ms) and highly significant in the defective parts of the visual field (RT-pre: 531.7 ± 13.4 ms, RT-post: 449.9 ± 19.5 ms; Wilcoxon test: *Z* = 2.90, *p* = 0.004; Figure 3D). RT improvements were most pronounced in areas of residual vision.

As was the case for DPR thresholds, the reduction of RTs during training was independent of the eccentricity in the visual field (MANOVA: df = 4, *F* = 0.58; *p* = 0.98). However, the pre-training light detection performance of the respective visual field position largely predicted the amount of improvement (Figure 4B), i.e., like in DPR thresholds the improvement was influenced by defect depth (MANOVA: df = 4, *F* = 12.79; *p* < 0.001).

Patient 7 who did not have a large transition zone showed no significant improvement of RTs over the treatment period (RT-pre: 391.0 ± 3.8 ms, RT-post: 375 ± 8.1 ms; Wilcoxon test: *Z* = 1.48, *p* = 0.138, Figure 2), and his RTs were not significantly different from those of the healthy sample, neither before nor after training. Patient 4, in contrast, was significantly slowed in his reaction to simple light stimuli when compared to age-matched subjects with normal vision. Performance remained lower than normal after the training, although his RTs significantly improved during treatment (RT-pre: 682.6 ± 32.8 ms, RT-post: 527.0 ± 8.4 ms; Wilcoxon test: *Z* = 2.02, *p* = 0.043, Figure 2). Patient 9 who improved considerably in his light detection performance during training also showed a pronounced reduction of his RTs by 45 ms (RT-pre: 423.8 ± 20.2 ms, RT-post: 379.1 ± 6.8 ms; Wilcoxon test: *Z* = 3.15, *p* = 0.002). The RTs in the previously blind field reached the level of the intact field before training (Figure 2). Overall, however, patient 9’s RTs were much longer than those of age-matched healthy controls which may be due to an impairment of the motor component of reacting to the light stimuli which did not improve as a result of the treatment.

Before training, the mean RTs of all patients were significantly longer than in the healthy sample (RT patients/pre: 484.8 ± 37.6 ms, RT healthy: 362.3 ± 3.5 ms; Mann–Whitney test: *Z* = 12.37, *p* < 0.001). RTs were slightly longer in the defective region of the visual field than in the patients’ intact regions, though the difference was not significant due to the high variance. Even RTs in the intact area of the patients were significantly longer than in the healthy group, which may also be due to a general slowing of RTs due to the brain lesion (see Discussion; RT patient/intact: 448.2 ± 83.6 ms; RT healthy: 362.3 ± 67.1 ms; *Z* = –9.58, *p* < 0.001) After treatment, patients’ RTs were, on average, still significantly longer than those of the healthy age-matched controls (RT patients/post: 452.4 ± 26.5 ms, RT healthy: 362.3 ± 3.5 ms; Mann–Whitney test: *Z* = 9.57, *p* < 0.001), but a few patients reached the level of normal subjects or even had normal RTs before training (see patient examples above and Figure 2).

Before training, DPR thresholds and RTs were highly correlated in the patient sample (Spearman’s Rho = 0.98, *p* < 0.001). This correlation was much reduced after training (Rho = 0.64, *p* = 0.09).

## DISCUSSION

Based on previous studies with healthy subjects and patients suffering from partial blindness, we wished to learn whether a restorative treatment designed to improve light detection would also change temporal perceptual performance in patients with visual field loss after brain lesions. In case we would find such a generalization of training effects, the question further was whether the level of improvement would reach that of age-matched healthy controls.

The study presented here was based on a solid body of psychophysical measurements of light detection and temporal processing with high spatial detail and the opportunity to do point-by-point comparisons in the visual field. Moreover, since our methodology was identical to our previous studies, the patient data could be directly compared to normative data of a healthy sample from the same age group.

### LIGHT DETECTION AND TEMPORAL PROCESSING

How temporal processing of visual signals is achieved, and how light detection and other basic visual functions are connected with temporal variables, is largely unknown. Evidence from studies with healthy participants points to apparently close connections between visual stimulus intensity on the one hand, and temporal visual functions (e.g., RTs, flicker detection) on the other hand (e.g., Kelly, 1972; Ulrich et al., 1998). However, these findings are based on single-point, often exclusively foveal, measurements which are not representative of the whole visual field (Poggel and Strasburger, 2004; Strasburger et al., 2011; Poggel et al., 2012a,b), i.e., the spatial dimension of vision is mostly neglected. In addition, flicker detection tasks suffer from various methodological problems like dependence on adaptation and on modulation depth (Tyler, 1985, 1987; Treutwein, 1989; Tyler and Hamer, 1990; Treutwein and Rentschler, 1992).

In more recent years, methods have been developed that allow topographical testing of temporal variables in patients with vision loss. For example, component perimetry (Bachmann and Fahle, 2000) simultaneously presents stimuli of a certain category (e.g., dynamic patterns) across the visual field and tests subjective perception in the defect area. This method is well suited for a rapid detection of visual field defects but does not provide a detailed map of visual thresholds. Various approaches of flicker perimetry (Rota-Bartelink, 1999; McKendrick, 2005) also allow detailed topographical threshold testing. Their clinical application is mostly targeted at retinal or other eye diseases, but they have not yet been systematically applied for the examination of patients with post-geniculate defects.

In the present study, we employed measurements of DPR and of RTs in a topographical fashion and directly compared their topographical patterns to those of perimetric and campimetric measures of light detection performance. DPR thresholds are more reliable than flicker detection thresholds because (a) the technique avoids dependence on adaptation and on modulation depth (Tyler, 1985, 1987; Treutwein, 1989; Tyler and Hamer, 1990; Treutwein and Rentschler, 1992), and (b) targets and non-targets appear equally bright since they are well above the summing duration in Bloch’s law (Treutwein, 1989; Treutwein and Rentschler, 1992). DPR thresholds also have the advantage of being independent of motor responses, in contrast to RTs (Schiefer et al., 2001; Poggel and Strasburger, 2004).

While forced-choice measurement of thresholds is more time-consuming and puts higher demands on the patient than do conventional clinical methods, the resulting measures are much more robust, free of observer bias, and allowed us to show—for the first time—in detail in how far the topographical patterns of variables of light detection and temporal processing overlap. In addition, the use of two different temporal variables (RTs and DPR thresholds) is useful to disentangle motor and visual components of processing speed.

To examine potential overlap or dissociations of light detection and temporal processing performance across the visual field, we had earlier used the tools described above to characterize a large sample of healthy subjects between 10 and 90 years of age (Poggel et al., 2012a,b). Unexpectedly, we had found that the visual field maps of perimetric thresholds, of RTs, and of DPR thresholds not only showed quite different topographic patterns, but also that the three variables showed different topographic patterns of aging. Hence, there is a dissociation of light detection and temporal variables both across the visual field and across the life span.

Another strategy to elucidate connections or dissociations between visual functions is their measurement in the damaged visual system. Here it is possible to check if loss of one function (detection) is associated with or dissociated from loss of another function (temporal processing). Patients with lesions of the visual pathway typically suffer from visual field defects, i.e., a loss, or reduction, of light-detection performance in a circumscribed region of the visual field. There is some evidence for a dissociation of perimetric thresholds and the topography of letter-contrast thresholds as well as RTs in patients with visual field loss (Strasburger and Rentschler, 1996; Gothe et al., 2000; see also Bola et al., 2013b, for a review). Hence, a topographic mismatch between different visual functions might explain why some forms of visual impairment remain undetected in clinical testing. In fact, many patients with visual field defects complain about difficulties of visual perception that escape detection with perimetric testing or other common measures of visual function. Frequently, these complaints are simply discarded as groundless (Poggel, 2002). While standard visual diagnostics are mainly concerned with the intensity aspect of vision (as assessed by perimetric luminance thresholds), the temporal dimension is usually neglected. Thus, some of the patients’ subjective complaints may be the result of temporal processing deficits which are not included in routine clinical testing.

To achieve a detailed comparison of light detection and temporal variables across the visual field in patients with damage to the visual pathway, we previously investigated a patient sample (Poggel et al., 2011) with the same methods described above (Poggel et al., 2012a,b). Compared to healthy subjects, DPR thresholds turned out to be elevated, and RTs were increased in the patients’ entire visual field, including areas that were perimetrically intact. Performance on temporal variables within the defective visual field depended on the degree of intactness of the respective visual field location. However, whereas DPR thresholds were increased around blind regions relative to the intact field, this was not the case for RTs. Thus, temporal processing in patients with cerebral vision loss is also impaired, but to a certain extent temporal processing appears to happen independently from perimetric light detection performance. This may partly explain reported subjective perceptual problems. The increased RT level in perimetrically intact areas was also confirmed in other samples of patients with pre- and post-geniculate damage to the visual system (Bola et al., 2013a; Sabel and Gudlin, 2014).

### PERCEPTUAL LEARNING AND VISION RESTORATION TRAINING

The overlap or dissociation of visual functions is of considerable interest for several reasons: the findings of studies with normally sighted and visually impaired populations are important for explaining basic mechanisms of visual processing in the healthy and the damaged visual system, i.e., how visual and temporal processing are connected (or disconnected) in the brain. Secondly, the results provide important information on the usefulness of diagnostic procedures, e.g., the fact that perimetric measurements are often not sufficient for obtaining a complete picture of the patient’s visual problems. A third important aspect concerns the therapeutic domain and processes of visual brain plasticity.

Human studies on perceptual learning in healthy subjects (Fine and Jacobs, 2002; Seitz and Watanabe, 2005; Jüttner and Rentschler, 2008; Fahle, 2009; Gilbert et al., 2009; see Strasburger et al., 2011 for review) showed that visual performance and hence visual brain areas are plastic throughout the life span. The observed improvements are usually specific to a visual function or to the visual field region targeted by the training (Fine and Jacobs, 2002; Fahle, 2009; Strasburger et al., 2011) and show only little, if any, generalization.

Similarly, clinical studies with patients suffering from vision loss after lesions to the visual pathway (for example van der Wildt and Bergsma, 1997; Kasten et al., 1998; Kerkhoff, 1999; Sabel, 1999, 2008; Poggel, 2002; Julkunen et al., 2003; Poggel et al., 2004; Sahraie, 2007; Huxlin, 2008; Bergsma and van der Wildt, 2010) have demonstrated training-induced improvement of function, particularly of light detection performance (see Sabel et al., 2011, for review). Despite earlier criticism (Pambakian and Kennard, 1997; Reinhard et al., 2005), there is substantial evidence that a partial restoration of visual function is possible in quite a number of patients (about one third showing either large, small, or no improvement, respectively) and that the training effect cannot be simply explained as being artifactual, like stemming from eye movements (Sabel et al., 2005; Kasten et al., 2006) or observer criterion shift (Poggel, 2002; Poggel et al., 2004). Similar to perceptual learning experiments with normally sighted samples, training studies targeting the improvement of light detection in patients with vision loss showed only little generalization to other functions like color and form discrimination (Kasten and Sabel, 1995; Kasten et al., 2000): a specific training of that particular function had a much more pronounced effect (Poggel, 2002).

### IMPROVEMENT OF LIGHT DETECTION PERFORMANCE AND TEMPORAL PROCESSING VARIABLES

#### Improvement of Light Detection Performance

The results presented in this study replicated earlier studies with respect to campimetric light detection improvement, i.e., increase of intact visual field size in patients with cerebral vision loss (Kasten and Sabel, 1995; van der Wildt and Bergsma, 1997; Kasten et al., 1998; Poggel et al., 2004; Sahraie, 2007; Huxlin, 2008; Bergsma and van der Wildt, 2010; but see Reinhard et al., 2005; Schreiber et al., 2006; for review see Sabel et al., 2011) and patients with pre-chiasmatic lesions of the visual system (Kasten et al., 1998; Sabel and Gudlin, 2014). Despite shorter daily training sessions (15 instead of 30 min) and a shorter treatment period of 3 instead of 6 months in the current study, the average extent of visual field increase was comparable to that of earlier studies, as was the considerable variability of training outcome in individual patients. These findings had been expected based on an earlier analysis of predictors of training outcome (Poggel et al., 2008).

Improvement in the high-resolution computer-based visual field test (HRP) was also confirmed by a significant decrease of the number of absolute defects in conventional perimetry, the established standard of visual field measurement.

#### Improvement of Temporal Resolution (DPR Thresholds)

For the first time we have now shown that a training regime designed to improve light detection generalizes in its effects to an improvement of temporal-resolution thresholds, i.e., to a function not specifically trained during treatment. Importantly, DPR thresholds are independent of motor responses, i.e., neither the elevated DPR thresholds nor their improvement during the training period can be explained by the patient’s motor function.

Interestingly, the improvement of DPR thresholds was significant only in transition zones, i.e., the areas between intact and blind visual field regions. These areas of residual vision are the crucial regions where the increase of light detection takes place, and their size has been shown to be the best predictor for training success out of a large number of relevant parameters that were tested (Kasten et al., 1998; Poggel et al., 2004, 2008). The findings suggest that basic visual processes like simple light detection and temporal resolution may be closely connected functionally and also in terms of neural-network connectivity and plasticity. This view is also supported by some topographical similarity of DPR and perimetric threshold maps in healthy subjects (Poggel et al., 2012a,b). We argued earlier that the detection of a temporal gap between the two light pulses during DPR threshold measurement requires a (possibly early cortical) readout mechanism that would detect and encode the drop in luminance within the double-pulse stimulus (Poggel et al., 2006). Thus, DPR thresholds seem closely linked to early levels of light perception (Fain and Cornwall, 1993) and may be improved when light detection thresholds are restored in a particular region of the visual field. This account is also supported by the observation that DPR improvement depended on the functional status (i.e., light detection probability or degree of impairment) of a particular region before training. Those areas with the greatest potential for an increase of light detection also exhibited the largest decrease of DPR thresholds over the training period.

The inter-individual variability of DPR training effects was considerable, however: some patients showed practically unchanged levels of temporal resolution before and after treatment, while others improved significantly. Of the latter, not all reached the level of healthy subjects in their age group and retained some residual impairment (see examples in Figure 2). Hence, in some patients the topographical improvement of DPR and light detection was almost entirely overlapping, while in others there was a clear topographical dissociation between functional restoration of those two parameters. From studies with patients suffering from right-parietal lesions (e.g., Battelli et al., 2003), one might conclude that an influence of higher visuo-cognitive functions on temporal processing (e.g., onset and offset detection of flickering stimuli) might be an explanation for the differences found between our patients. However, in our—admittedly small—sample we could not find systematic effects of the hemisphere affected by the lesion, the lesion size (as estimated by the size of the blind area), or the location of the lesion in the region perfused by the posterior or middle artery. Furthermore, both visuo-spatial neglect and higher-order visual or cognitive deficits were exclusion criteria. A detailed lesion analysis in larger patient groups needs to be carried out to test the assumption that in patients with a dissociation of light-detection and temporal-performance measures, additional brain areas are affected that would normally coordinate performance (and possibly also functional recovery). Here we can only conjecture that connections to fronto-parietal networks may play a role in top-down coordination of light detection and temporal visual performance. Recent evidence points to changes in brain connectivity taking place during vision restoration (Bola et al., 2014).

#### Improvement of Simple Reaction Times

In several studies, elevated levels of RTs both in the intact and in the defective parts of the visual field in visually impaired patients have been confirmed (Poggel et al., 2004; Mueller et al., 2007; Bola et al., 2013a). This effect is found both in patients with pre-geniculate as well as with post-geniculate damage to the visual pathway. The increase of RTs depends both on local factors (the proximity to the scotoma in the individual patient’s visual field) and on global factors (the size of the blind area, with longer RTs found in patients with larger scotoma; Bola et al., 2013a). While RT to simple light stimuli (as measured here using high-resolution campimetric testing, HRP) depend not only on visual temporal processing but also on the speed of the motor response, this is only true for the average RT value in a patient’s result: the variation of RTs across the visual field reflects the sensory component only since motor requirements are invariant, i.e., contribute to the absolute level of RT only (Teichner and Krebs, 1972; Schiefer et al., 2001).

As already shown in previous research (Poggel, 2002; Poggel et al., 2004; Mueller et al., 2007), simple RTs to the detection of light stimuli also improved significantly during restoration training. This was recently also shown in patients with glaucoma (Sabel and Gudlin, 2014). Very likely, the improvement of RTs (and also of DPR thresholds) in response to restoration training is not specific for VRT, but should be a “positive side effect” of any method suitable for improving light detection performance. Although in our sample the improvement of RTs was small across the whole of the visual field, it was pronounced and highly robust in areas of residual vision around the blind parts of the visual field. Again, the functional status of a specific visual field position mainly determined whether, and to what extent, RT improvement was observed during treatment. The overall decrease of RTs in transition zones was closely connected to each patient’s pre-training performance. Still, many patients remained at a level of severely increased RT compared to normal subjects even after training. Regions of elevated RTs remarkably included the perimetrically intact areas. Therefore, most of this residual impairment was likely due to unspecifically longer motor RT resulting from the cerebral damage. Since perimetric testing included contrast threshold measurements which were normal in the patients’ intact visual field regions, increased RT levels in the perimetrically intact parts of the visual field could not be explained by deficits of contrast perception in intact areas (Plainis and Murray, 2000).

### CONCLUSION

In summary, our findings show that the examination of temporal parameters of visual perception, in addition to spatial information processing, helps explain residual visual impairment that cannot be detected by exclusively using standard perimetric testing. Moreover, using our detailed maps of temporal functions, the improvement of dynamic characteristics of vision can be followed during recovery of vision, either spontaneous or induced by training. Further research on the relationship of basic visual functions and temporal functions will be required to more fully understand their interactions. In the current study we only show the relation between detection and temporal-processing performance, but it would be interesting to test cross-modal effects to obtain insight into potential supra-modal aspects of temporal processing or changes of temporal processing during perceptual learning. Also, an investigation of a larger sample of patients allowing for a detailed lesion analysis would be necessary to be better able to understand the influence of lesion size and location on temporal deficits and their recovery during training. An important question arising from our findings is whether, and to what extent, patients with selective impairments in the temporal domain of vision can be helped using a specific training for temporal aspects of vision. Hence, by gaining more knowledge about the interaction of light detection and temporal functions of vision we will be able to design more efficient techniques of vision restoration.

### Conflict of Interest Statement

At the time the study was conducted, Dr. Bernhard A. Sabel was shareholder of NovaVision Inc., the manufacturer of VRT..
